# Immunotherapy in Melanoma, Gastrointestinal (GI), and Pulmonary Malignancies

**DOI:** 10.3934/publichealth.2015.1.86

**Published:** 2015-03-24

**Authors:** Alexander B. Dillon, Kevin Lin, Andrew Kwong, Susana Ortiz

**Affiliations:** Mount Zion Cancer Research Center, Department of Dermatology, University of California San Francisco, CA 94141, USA

**Keywords:** immunotherapy, melanoma, esophageal cancer, gastric cancer, colorectal cancer, lung cancer, IL-2, adoptive cell therapy, anti-CTLA-4, anti-PD-1

## Abstract

Oncologic immunotherapy involves stimulating the immune system to more effectively identify and eradicate tumor cells that have successfully adapted to survive the body's natural immune defenses. Immunotherapy has shown great promise thus far by prolonging the lives of patients with a variety of malignancies, and has added a crucial new set of tools to the oncologists' armamentarium. The aim of this paper is to provide an overview of immunotherapy treatment options that are currently available and under active research for melanoma, gastrointestinal (esophageal, gastric, pancreatic, and colorectal), and pulmonary malignancies. Potential biomarkers that may predict favorable responses to immunotherapies are discussed where applicable, as are future avenues of research in this rapidly evolving field.

## 1. Introduction

The field of cancer immunotherapy was born in the late 1800s when a notable discovery of a patient's tumor regression following a bacterial infection led American physician William B. Coley to intentionally elicit anti-tumor immune responses in patients with subcutaneous and intralesional injections of heat-killed *Streptococcus pyogenes* and *Serratia marcescens* bacteria, coined “Coley's toxins” [1],[2]. Over 120 years later, a number of life-prolonging therapeutic advances and the quest for more individualized care have propelled immunotherapy to the forefront of the battle against cancer. Current immunotherapeutic approaches include 1) use of exogenous cytokines to non-specifically stimulate the immune system's effector cells to mount an anti-tumor response, 2) introduction of immuno-stimulatory antigens to precipitate a targeted immune response (i.e. active immunization or tumor vaccination), 3) exogenous expansion and reinfusion of tumor-specific immune cells (adoptive immunotherapy), 4) immune system checkpoint modulation, and 5) use of cancer-killing and immune system-stimulating modified viruses (oncolytic immunotherapy). Using this framework, the aim of this review is to provide an overview of the latest advances in this field as they pertain to melanoma, gastrointestinal, and pulmonary malignancies. To this end, the authors conducted a thorough review of PubMed, the National Institute of Health's ClinicalTrials.gov, the American Society of Clinical Oncology (ASCO), and the European Society for Medical Oncology (ESMO) databases.

## 2. Melanoma

Melanoma, cancer derived from melanocytes, has earned a reputation as one of the most immunogenic tumors due to the observations that primary lesions often elicit lymphocytic infiltration that may lead to their partial or complete regression [3], development of autoimmune depigmentation portends a better prognosis [4], and immunotherapies have yielded significant, life-prolonging results [5]–[8]. For decades, the treatment of metastatic melanoma (MM) relied largely on chemotherapeutic agents, particularly dacarbazine and its oral analog temozolomide, and yielded modest outcomes, especially in disseminated disease, with 5–15% response rates, median overall survival (OS) of 6–10 months, and 5-year survival rates of ∼10% [9],[10]. The introduction and approval of multiple systemic immunotherapies over the past decade, however, have begun to favorably transform the treatment and outcome landscape (Table 1).

### 2.1. Immunostimulatory cytokines

#### 2.1.1. Interferon alpha (IFN-α)

In 1995, IFN-α, a protein involved in immunomodulatory cell-cell signaling, including MHC I-based antigen recognition, chemokine production and secretion, and immune cell activation, among other anti-tumor properties [11], became the first and only FDA-approved adjuvant therapy for post-resection, high-risk MM. In this capacity, high dose IFN-α (HD IFN-α) is employed as a means of minimizing the risk of recurrence after all detectable regional disease has been surgically removed.

**Table 1.** FDA-approved melanoma immunotherapies.

Strategy	Agent	Class	Mechanism of Action	Year of FDA approval	Monotherapy vs. Adjuvant	Target Patient Population	Route(s)/ Regimen(s)	Predictors of Favorable Response Rates	Notable Adverse Drug Reactions (ADRs)
Non-specific stimulation of immune system effector cells	Interferon-alpha (IFN-α)	Cytokine Protein	Directly inhibits tumor cell proliferation. Enhances innate & adaptive immunity. Facilitates tumor antigen recognition via enhanced MHC I receptor expression. Represses oncogenes and induces tumor suppressor gene expression. Inhibits angiogenesis.	1995 (IFN-α) 2011 (pegylated IFN-α2b)	Adjuvant	Stage II-III resected melanoma patients with good performance status and no evidence of psychiatric or autoimmune disease	20 MU/m^2^ IV daily × 1 mo, followed by 10 MU/m^2^ SC TIW × 1 yr	IFN-α: micrometastatic disease peg-IFNα: ulcerated primary lesions, micrometastatic disease	Hepatotoxicity
	High-Dose Interleukin 2 (HD IL-2), Aldesleukin	Cytokine Protein	Activates B, T, & NK cells, facilitating cytolytic destruction of tumor cells	1998	1st-line monotherapy	Intravenous: stage IV BRAF wild-type melanoma patients with good performance status and no evidence of CNS disease Intralesional injections: inoperable in-transit metastases	Intravenous: 600–720k IU/kg q8h × 14 consecutive doses over 5 d, repeat w/in 6–9 d Intralesional: 0.3–18 mm IU each lesion SC 2-5x/wk	NRAS mutations, limited SC or cutaneous metastatic disease, absence of elevated serum LDH, VEGF, fibronectin, and CRP	Hypotension, pulmonary and systemic edema, renal insufficiency Intralesional injections minimize systemic toxicity
Modulation of immune system checkpoints with monoclonal antibodies (mAbs)	Ipilimumab	Anti-CTLA-4 mAb	Blocks CTLA-4 receptor on tumor cells, thereby circumventing downregulation of the T cell response	2011	Monotherapy in unresectable and/or metastatic disease Adjuvant therapy in resectable disease at high risk of recurrence		10 mg/kg IV q3W × 4 doses, then q12W maintenance	Baseline high tumor cell expression of FOXP3 and indolamine 2,3 dioxygenase (IDO) as well as high tumor infiltrating lymphocyte count	GI toxicity including colitis, hepatotoxicity, nephrotoxicity, thyroid toxicity
	Nivolumab	Anti-PD-1 mAb	Blocks PD-1 receptor on immune effector cells, circumventing downregulation of the cellular immune response	2014	2nd-line monotherapy	Metastatic melanoma, refractory to CTLA-4 and/or BRAF inhibition	3 mg/kg IV q2W	Higher PDL-1 expression on tumor cells	Colitis, hepatoxicity, pulmonary toxicity, nephrotoxicity, thyroid toxicity
	Pembrolizumab (MK-3475)	Anti-PD-1 mAb	Blocks PD-1 receptor on immune effector cells, circumventing downregulation of the cellular immune response	2014	2nd-line monotherapy	Metastatic melanoma patients post-treatment with ipilimumab or combination ipilimumab and BRAF inhibition in those with BRAF-mutated tumors	2 mg/kg IV q3W	Higher PDL-1 expression on tumor cells	Cellulitis, sepsis, renal failure, pneumonia, GI toxicity, anemia

The FDA's approval followed a 287-patient randomized phase III trial demonstrating significantly prolonged relapse-free and overall survival (median RFS 1.7 yrs vs. 1 yr, *p* = 0.002, median OS 3.8 vs. 2.8 yrs, *p* = 0.023) with adjuvant treatment post-resection at maximum tolerated doses (20 MU/m2 IV daily × 1 mo, followed by 10 MU/m2 SC TIW × 1 yr) compared with observation over a median 6.9 year follow-up period [12]. The difference in outcomes was most pronounced for patients with microscopic but not clinically apparent nodal disease. Toxicity was significant, with more than two-thirds experiencing grade III-IV adverse drug reactions (ADRs), and two deaths from hepatotoxicity. Subsequent randomized studies and meta-analyses evaluating varying dosages and treatment durations have not convened on any alternative recommendations, though they generally agree upon a statistically significant improvement in RFS (level A evidence), with a more modest impact on OS [13],[14]. The Society for Immunotherapy in Cancer (SITC) recommends the 1-year regimen as an adjuvant option for stage II–III patients with good performance status and no evidence of psychiatric or autoimmune disease [15].

In 2011, the FDA approved pegylated IFN-α, reducing its immunogenicity and increasing the agent's half-life by reducing its absorption rate following subcutaneous injection, as well as its clearance [16]. Long-term (median 7.6 yr) follow-up data demonstrated a significant survival benefit in a subset of patients with ulcerated primary lesions and micro-metastatic disease (HR = 0.58, *p* < 0.0001, level B evidence) [17]. A number of other promising prognostic biomarkers that might be used to narrow the target patient population and maximize the therapeutic threshold have since been identified (e.g., serum TNF-α, β-2 microglobulin, and sIL-2R), though none are yet adequately validated or widely clinically used [14],[18].

Comparisons of HD IFN-α to biochemotherapy (chemotherapy + low dose IFN + IL-2) have demonstrated no significant difference in OS, and increased toxicity with the latter [19],[20]. New combination therapies are also currently under investigation. For example, based on preclinical data suggesting synergy between IFN-α and BRAF inhibitors in BRAF-mutated disease, two phase I/II clinical trials are currently evaluating the combination of IFN-α/peg-IFN-α and vemurafenib in BRAF-mutated MM (NCT01943422, NCT01959633).

#### 2.1.2. Interleukin-2 (IL-2)

Three years after the approval of IFN-α, the FDA approved the use of high-dose interleukin-2 (HD IL-2) as the first mono-immunotherapy in MM. IL-2 is naturally produced by T cells and it activates B cells and macrophages and facilitates the cytotoxicity of T and natural killer (NK) cells. The SITC currently endorses the use of recombinant HD IL-2 as first-line therapy for stage IV MM patients with good performance status and no active CNS disease [15]. The generally accepted dosing regimen is 600–720 IU/kg IV over 15 min, q8h up to a maximum of 14 consecutive doses, with a second cycle following a 6–9 day hiatus [21],[22]. Objective response rates from randomized and phase II clinical trials have ranged from 5–33%, and complete responses from 0–15%, and while no clear overall survival benefit has been demonstrated, the subset of complete responders in phase II trials have enjoyed median long-term responses of > 5 years (range 1.5 mo–12.3 yrs) [21],[23]. Toxicity can be significant and acute, ranging from nausea, vomiting, and diarrhea to hypotension and renal and hepatic dysfunction, though it is generally transient and reversible. Death rates from toxicity range from ∼1.7–3.7% [23]. Patients most likely to respond favorably include those with good performance status, limited cutaneous or subcutaneous disease, normal serum LDH, and NRAS mutations [24],[25]. The use of HD IL-2 is also being investigated in conjunction with other systemic immunotherapeutic agents. For example, a phase IV clinical trial assessing the efficacy of HD IL-2 in combination with vemurafenib is currently recruiting (NCT01683188).

When surgery is not an option for in-transit metastases (those between the primary lesion and the draining lymph nodes), intralesional and subcutaneous injections of IL-2 of varying regimens (0.3–18 mm IU 2–5 x/wk for 1–53 weeks) have yielded average complete response rates of ∼50% (range 0–69%) with significantly less toxicity than systemic treatment, and provided complete responders with greater OS than partial responders [26],[27]. Efforts to artificially increase the body's expression of interleukins with gene therapy via DNA plasmid vectors and electroporation have also been explored with some success, particularly in the case of IL-12. A small (*n* = 19) phase I trial showed a partial, disease-stabilizing response in 42% of MM patients and a complete response, including resolution of distant metastases in the absence of systemic therapy, in 10% [28]. A corresponding phase II trial is currently recruiting (NCT01502293).

### 2.2. Anticancer vaccines

At present, there are no FDA-approved tumor vaccinations for melanoma. The aim of this technique is to facilitate tumor antigen recognition and a subsequent anti-tumor immune response by artificially introducing tumor-associated antigens to the body, or cellular equipment that can help expose those already present. Artificially introduced antigens can take the form of peptide fragments, whole proteins, cell lysates or whole cells. While use of these agents, alone, or in combination with non-specific immuno-adjuvant therapies like HD IL-2, has not revealed a survival advantage, active specific therapy, that is employment of autologous dendritic cells (DCs) as vectors for tumor-specific antigens, has. A 42-patient, randomized phase II trial comparing the efficacy of DCs loaded with autologous tumor stem cell content to irradiated tumor stem cells alone showed a significant survival benefit with the former over median follow-up periods of 15–24 months (range 6–47 mo, HR 0.27, 95% CI: 0.098–0.729) [29]. Two-year survival rates were 72% and 31% (*p* = 0.007). Pooled data from this study and that of two other single-arm phase II trials revealed a median overall survival of 38.8 mo in the DC vaccine group compared to 14.7 mo for the control group and a 5-year OS of 33% vs. 20% (HR = 0.65, *p* = 0.025) [30].

Future approaches include RNA vaccines (phase I, NCT01684241, NCT02035956), DNA vaccines (phase I/II, NCT01138410), and combination therapy with peptide vaccines and anti-CTLA-4 checkpoint blockade (phase I, NCT01810016). Randomized phase II clinical trials are also exploring combinations of DC vaccines with radiation therapy and pre-vaccination IFN-α (NCT01973322), modified tumor cell-based vaccines with ipilimumab (NCT02054520), and DC vaccines with targeted tyrosine kinase inhibitors (NCT01876212).

### 2.3. Adoptive cell transfer

Adoptive cell therapy (ACT) involves harvesting autologous lymphocytes from a patient's tumor or peripheral blood, expanding them and possibly modifying them in-vitro to express tumor-associated antigen receptors or secrete specific cytokines, and reintroducing them back into the host. This procedure is often preceded by high-dose, lympho-depleting chemotherapy to minimize immune regulatory elements, and thus maximize the anti-tumor response. In the context of the small, largely unrandomized studies to date, ACT has not yet demonstrated a clear survival benefit in MM patients, and due to the labor and resources it involves, as well as the toxicity associated with myeloablative pre-conditioning, it is currently reserved for the experimental treatment of disease refractory to 1st-line systemic treatment. Multiple phase II trials have achieved promising results with objective overall response rates (ORR) as high as 72% following more intense pre-condition with chemotherapy and total body radiation, 22% complete response (CR) rates, and 3- and 5-year OS of 36% and 29% (100% and 93% in the subgroup of complete responders) [6],[31]. Short culture time, youth and CD8 positivity of the tumor infiltrating lymphocytes (TILs) used, and high TIL number have all correlated with better ACT responses [32],[33], though the need for further predictive prognostic biomarkers remains due to the financial, temporal, and toxicity-related burden associated with this treatment technique.

Future ACT approaches include combination therapy with DC vaccination (phase I, NCT01946373), as well as with BRAF inhibition and HD IL-2 (phase II, NCT01659151). A phase III randomized trial comparing peripheral blood monocyte ACT with an autologous tumor stem cell-loaded DC vaccine is also recruiting (NCT01875653).

### 2.4. Immunomodulatory monoclonal antibodies (mAbs)

#### 2.4.1. Cytotoxic T-Lymphocyte Antigen-4 (CTLA-4) inhibition

**Figure 1.**
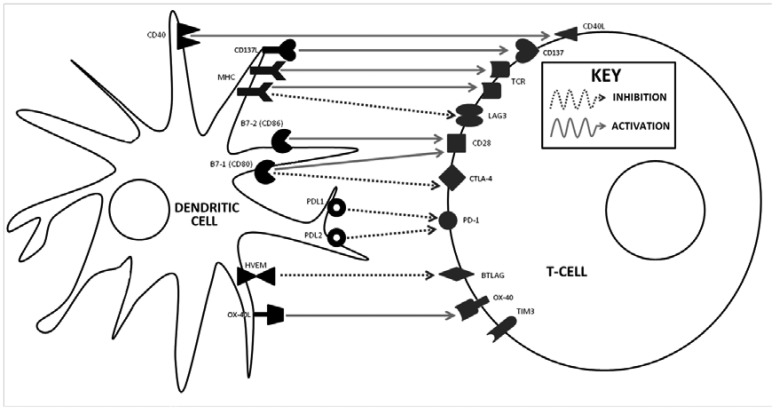
Dendritic and T cell interactions and targets of immunomodulatory mAbs. *Reproduced with permission from Sanlorenzo et al*.

An increasingly detailed understanding of the molecular checks and balances of immunology paved the way for the development of the latest class of immunotherapeutics, immune-checkpoint modulators. In 2011, the FDA approved the first such agent for MM, ipilimumab, a mAb antagonist of the Cytotoxic T-Lymphocyte Antigen-4 (CTLA-4) receptor. CTLA-4 is a T cell receptor that naturally interacts with B7-1 (CD-80) and B7-2 (CD-86) on the surface of antigen presenting cells, thereby down-regulating the T cell response and avoiding potential autoimmune damage (Figure 1). A costimulatory T cell surface protein, CD-28, on the other hand, competes with CTLA-4, albeit with less affinity, for interaction with B7-1 and B7-2, activating the T cell [34]. Blocking CTLA-4 thereby allows CD-28 to interact with B7-1 and B7-2, enhancing the body's cellular immune response and ability to eradicate tumor cells. Ipilimumab's approval followed a 676-patient phase III randomized, controlled trial comparing ipilimumab (3 mg/kg IV q3W for up to 4 treatments) to a melanoma antigen-specific peptide vaccine, gp100, and the combination of the two in unresectable stage III-IV MM patients [35]. Median OS was significantly prolonged in the ipilimumab group than the gp100 group (10.1 mo vs. 6.4 mo, HR 0.66, *p* = 0.003), and the combination of the two did not yield any synergistic benefit. Grade 3–4 ADRs such as colitis occurred in 10–15% of ipilimumab-treated patients and in 3% of patients who received gp100 alone. Ipilimumab is now considered 1st-line therapy by the SITC in BRAF wild-type MM patients with poor performance status and/or untreated CNS disease [15].

Shortly thereafter, a phase II randomized dose-ranging trial found ipilimumab 10 mg/kg more effective than 3 mg/kg [36]. At this higher dose (10 mg/kg q3W × 4 treatments, then q12W for maintenance), a randomized, controlled phase III trial found ipilimumab plus dacarbazine (850 mg/m^2^) superior to chemo alone in 502 treatment-naive MM patients (OS 11.2 vs. 9.1 mo, 3-year survival 20.8% vs. 12.2%, HR 0.72, *p* < 0.001) [37]. Grade 3–4 ADRs were more frequent in the ipilimumab cohort (56.3% vs. 27.5%, *p* < 0.001). The durability of ipilimumab responses was Highlighted by multiple analyses of aggregated clinical trial data showing 5-year OS rates of 21.4–49.5% [38], and a survival plateau between ∼3–10 yrs for about one fifth of patients, even after a subset ceased treatment [7],[39]. Furthermore, retreatment with ipilimumab following disease progression yielded promising results in a recent study with 55% (28/51) regaining disease control and surviving a median of 21 months. Two-year survival was 42% post-reinduction, and ADRs were generally only mild-to-moderate in nature [40].

Comparisons of anti-CTLA-4 therapy to other treatments have been investigated, as have the efficacy of different treatment sequences including this checkpoint blockade. For example, a meta-analysis of 15 randomized trials demonstrated superior survival with ipilimumab monotherapy compared to chemotherapy alone, a variety of chemotherapy combinations, biochemotherapy, and other immunotherapies including peptide vaccines, IL-2, and IFN-α [41]. Anti-CTLA-4 treatment prior to BRAF inhibition has also been associated with a significant survival benefit over the reverse sequence in BRAF-mutated MM (*n* = 45, 14.5 mo vs. 9.9 mo), though median OS varied widely among the BRAF-ipilimumab cohort in this study depending on who did or did not complete the ipilimumab treatment protocol (12.7 vs. 1.2 mo, *p* < 0.001) [42].

**Figure 2.**
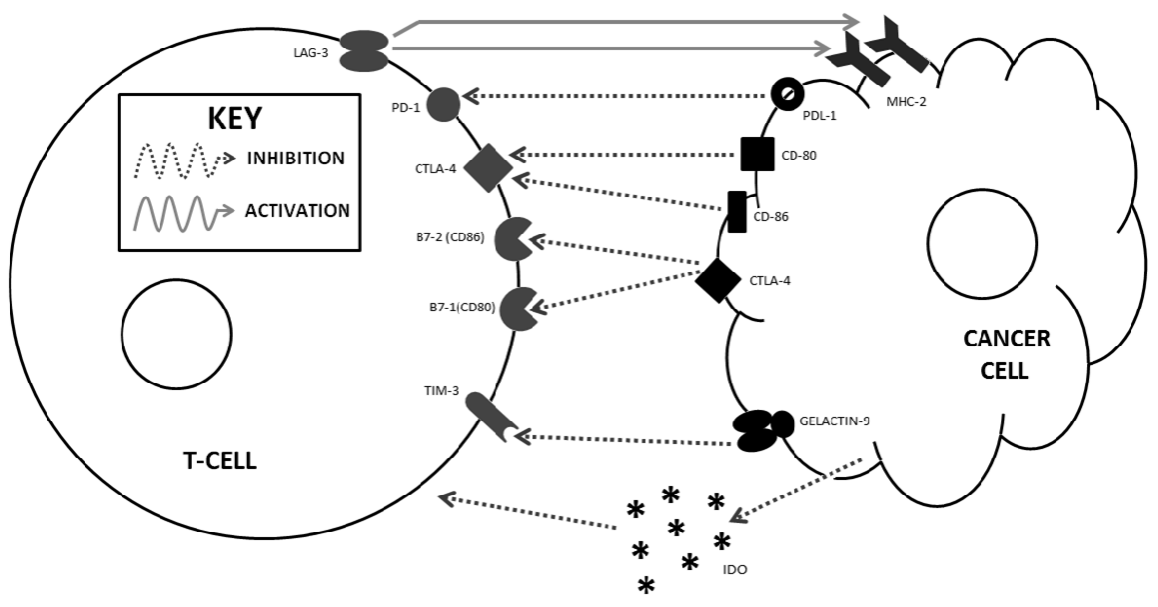
T cell and cancer cell interactions and targets of immunomodulatory mAbs. *Reproduced with permission from Sanlorenzo et al.*

Given the success of ipilimumab monotherapy, clinicians and researchers have been quick to explore combination therapies that might further improve outcomes. For example, a phase II randomized trial comparing ipilimumab (10 mg/kg) and systemic GM-CSF (sargramostim, 250 µg SC daily, d1-14) to ipilimumab alone in 245 MM patients, found that combination treatment conferred a greater OS (17.5 mo vs. 12.7 mo, HR = 0.64, *p* = 0.01) and a lower rate of grade 3–4 ADRs (44.9 vs. 58.3%, *p* = 0.04) [43]. A smaller, non-randomized trial showed evidence of a survival benefit with combination anti-CTLA-4 and radiation therapy (RT, *n* = 53) or steroids (*n* = 50) compared to CTLA-4 blockade alone (*n* = 83, *p* = 0.02 and 0.037, respectively) [44]. The latter finding is consistent with the well-documented observation that incidence and severity of immune-related side effects of immunotherapy correlate with response rate [45]. A phase II randomized trial is further investigating the anti-CTLA-4 and RT combination (NCT01689974), and another is exploring the efficacy of stereotactic RT followed by ipilimumab (NCT01497808). Other current avenues of research include combining CTLA-4 blockade and indolamine 2,3 deoxygenase inhibition (IDO, aberrantly expressed by tumors, breaks down tryptophan halting T cell growth, phase I/II, NCT01604889, Figure 2), as well as ipilimumab with ACT, with or without HD IL-2 (phase I, NCT01701674, NCT02210104).

Ipilimumab has also shown promise as an adjuvant to surgery in stage III MM patients at high risk of recurrence. For example, a randomized phase III comparison of ipilimumab (10 mg/kg, *n* = 475) to placebo (*n* = 476), demonstrated significantly improved RFS in the treatment group over 3 years (HR 0.75, 95% CI: 0.64–0.90), despite the fact that half of the cohort ultimately discontinued therapy due to ADRs (38.6% within the first 3 mo) [46]. The ADR-related death rate was 1.1%. Another randomized phase III trial comparing ipilimumab to HD IFN-α in the adjuvant setting is recruiting (NCT01274338).

Numerous potential prognostic biomarkers are under investigation to help guide clinical decision-making in an effort to maximize therapeutic efficacy of immune biologics and minimize unnecessary harm and expense. A randomized phase II biomarker study found that baseline tumor expression of FoxP3 and IDO correlates with clinical activity of ipilimumab, as does high TIL count [47]. An ongoing trial suggests germline genetic loci (e.g. RPS6KB1 and LNX2) may likewise serve as biomarkers for ipilimumab responses [48], though further investigation to uncover useful, easily measurable prospective biomarkers is warranted.

#### 2.4.2. Programmed Death-1 (PD-1) inhibition

In 2014, the FDA approved another duo of mAb immune checkpoint blockade agents, nivolumab and pembrolizumab, for the treatment of MM. Both medications target the Programmed Death-1 (PD-1) receptor expressed on B, T, and NK cells, which interacts with Programmed Death Ligands-1 and -2 (PDL-1 and -2), often subversively expressed on melanoma cells, to induce T cell exhaustion and down-regulate the immune response [49]. By blocking PD-1, these medications facilitate a more vigorous anti-tumor cellular immune response. Approval of nivolumab followed a randomized phase III trial comparing it (at 3 mg/kg IV q2W, *n* = 120) to investigator's choice chemotherapy (*n* = 47) in MM patients refractory to CTLA-4 and/or BRAF inhibition [50]. Overall response rates were higher in the nivolumab cohort (32% vs. 11%), and a reduction of at least 50% of the target tumor burden was achieved in 82% of nivolumab patients compared to 60% of the chemotherapy cohort. Furthermore, the nivolumab group experienced fewer grade 3–4 ADRs (9% vs. 31%). OS data is pending as of the publication of this manuscript.

Approval of pembrolizumab for patients with unresectable MM refractory to ipilimumab and BRAF inhibition (when applicable) was expedited after the outcomes of two trials. The first (KEYNOTE-001), a 135-patient phase I study, showed dose-dependent responses, with a peak ORR of 52% (95% CI: 38–66%) in the high-dose (10 mg/kg q2W) cohort, an overall median progression free survival (PFS) of 7+ mo, and a 23% rate of grade 3–4 ADRs [51]. The second, a randomized, international, multicenter expansion of the first, evaluated pembrolizumab's efficacy at two doses, 2 and 10 mg/kg q3W, in 173 MM patients refractory to two or more ipilimumab doses [52]. Both dosages yielded an ORR of 26%, ADRs (e.g., fatigue, pruritus, rash) were similar in frequency and mostly grade 1–2, and there were no treatment-related deaths. Recently, another randomized, dose-comparison expansion of KEYNOTE-001 found 10 mg/kg q3W (*n* = 121) comparable to 10 mg/kg q2W (*n* = 123, ORR 28% vs. 33%, PFS 43% vs. 47%, grade 3–4 ADRs 12% vs. 15%) [53]. As previously mentioned randomized data showed 2 mg/kg q3W comparable to 10 mg/kg q3W, 2 mg/kg q3W is now the recommended regimen.

Anti-PD-1 agents have already shown durable response rates. A pooled analysis of multiple pembrolizumab trials, including 411 MM patients with a minimum of 6 mo follow-up, demonstrated an ORR of 40% in ipilimumab-naive patients and 28% in ipilimumab-refractory patients, PFS of 23–24 weeks, 1-year OS of 71%, and a 12% incidence of grade 3–4 ADRs with only a 4% ADR-related discontinuation rate [54]. Likewise, a 13+ mo follow-up of a phase I study of 135 MM patients receiving pembrolizumab reported an ORR of 41%, with conversions to CRs seen as late as 1.5 years after treatment initiation, a median PFS of 7.75 mo, a 1-year OS rate of 81%, and a 14% incidence of grade 3–4 ADRs [55]. PDL-1 expression was found to correlate with ORR and PFS, though an anti-tumor response was still observed in patients with low PDL-1 expression. No drug-related deaths were reported in either study.

#### 2.4.3. Combination therapy

New avenues of research involve combining anti-PD-1 and anti-CTLA-4 therapy to realize possible synergistic benefits, comparing the two, and combining the former with other treatment modalities. For example, a recent phase I trial compared a combination of nivolumab and ipilimumab (*n* = 53) to sequential treatment with ipilimumab followed by nivolumab (*n* = 33) [56]. Concurrent therapy yielded on ORR twice that of sequential therapy (40 vs. 20%), and the generally reversible grade 3–4 ADRs followed suit (53 vs. 18%). The highest ORR was observed in the concurrent nivolumab 1 mg/kg, ipilimumab 3 mg/kg regimen (53%, 95% CI: 28–77%), and 41% (7/17) of these responders experienced at least an 80% tumor reduction at 3 months. A phase III trial is currently comparing nivolumab and ipilimumab head-to-head (NCT01844505).

Other lines of investigation include comparing pembrolizumab to chemotherapy (phase II, NCT01704287) and ipilimumab (phase III, NCT01866319), as well as evaluating pembrolizumab's efficacy in the neoadjuvant setting (phase II, NCT02306850). Preclinical data suggests RT may work synergistically with PD-1 blockade by upregulating tumor-associated antigen-MHC complexes and facilitating antigen cross-presentation in regional lymph nodes, thereby increasing antigen-experienced and effector memory T cells and CD8 TILs [57], representing another combination worthy of further investigation.

#### 2.4.4. PD-L1 inhibition and other potential checkpoint targets

Attempts to block Programmed Death Ligands with mAbs (BMS-936559 and MPDL3280A) have not yielded results as promising as PD-1 blockade in MM [58], though phase I trials are currently investigating their efficacy in combination with BRAF inhibitors (NCT01656642) and IFN-α (NCT02174172). In addition to CTLA-4 and PD-1, other immune checkpoints have arisen as potential targets of agonism and antagonism by mAbs including cell surface molecules whose stimulation promotes an anti-tumor T cell response such as CD40, and those, such as LAG-3, whose inhibition circumvents down-regulation of the cellular immune response when the body most needs it.

#### 2.4.5. CD40 agonism

CD40 is a costimulatory receptor of the tumor necrosis factor (TNF) family normally expressed on a variety of cells including dendritic cells and macrophages. Interaction with its ligand plays a key role in priming and proliferation of antigen-specific CD4 T cells. When expressed on tumor cells, its stimulation results in apoptosis [59]. Thus, CD40-stimulating mAbs (e.g., CD-870873) have direct anti-tumor activity and induce tumor antigen-specific T cell responses. So far, phase I data show an ORR of 27% (4/15 MM patients), with tolerable ADRs [60], and a preliminary partial response rate of 18.8% (3/16 patients) when administered in combination with chemotherapy (NCT00607048). An ongoing phase I study is exploring combination anti-CTLA-4 and CD40 agonist therapy in stage IV melanoma (NCT01103635), and another phase I/II trial assessing the safety and efficacy of CD40L gene therapy via a modified adenovirus vector is currently recruiting (NCT01455259).

#### 2.4.6. Lymphocyte Activation Gene-3 (LAG-3) and T cell Immunoglobulin Mucin-3 (TIM-3) inhibition

LAG-3 is a transmembrane protein expressed on T regulatory (T reg) cells that binds MHC II, often expressed on melanoma cells, thereby enhancing T reg activity, negatively regulating the cellular immune response, and protecting melanoma cells from apoptosis [61]. Blocking LAG-3 could thus help the body fight tumor cells on two fronts, and blocking PD-1 in conjunction, which also promotes immuno-tolerance of tumor antigens, might have a synergistic effect. A phase I trial evaluating this combination of mAbs in a variety of solid tumors is recruiting (NCT01968109). T cell Immunoglobulin Mucin-3 (TIM-3) is another T cell receptor often expressed on TILs in conjunction with PD-1, which, like PD-1 and LAG-3, also serves as a negative T cell regulator and thus a potential therapeutic target in MM, alone or in conjunction with anti-PD-1 therapy [62]. TIM-3 expression on NK cells has also been associated with their exhaustion in the setting of MM, and TIM-3 blockade has been shown to reverse this phenotype, providing further evidence to support this potential target [63]. No anti-TIM-3 clinical trials have yet begun.

#### 2.4.7. Toll-like receptor agonists

Another class of immunomodulators, including imiquimod (IMQ), FDA-approved in 2004 for the treatment of basal cell cancer, act upon Toll-like receptors (TLRs), a group of cell-surface receptors found on sentinel immune cells like dendritic cells and macrophages that naturally activate an innate immune response upon contact with characteristic pathogen-related antigens. Topical treatment of melanoma, with IMQ, a TLR-7 agonist, has been shown to facilitate 1) tumor infiltration with immune effector cells such as activated, cytotoxic plasmacytoid DCs, 2) a type I IFN response, 3) anti-angiogenic defenses, and in some cases result in complete tumor regression [64]–[66]. Preclinical data suggest that intralesional TLR-7/8 agonists can elicit antitumor responses both in injected and distant MM lesions, and may potentiate anti-CTLA-4 and anti-PD-1 therapy [67]. A number of phase I-II clinical trials are investigating the topical TLR-7/8 agonist resiquimod and/or injectable TLR agonists in concert with protein vaccines as adjuvant treatment for resected stage II-IV disease (NCT00821652, NCT00960752, NCT 02126579, NCT02320305).

### 2.5. Oncolytic viruses

Recently, a number of originally pathogenic viruses have been re-engineered to directly exterminate melanoma tumor cells and elicit a host immune response against distant tumor cells. While this technique is still in its early stages of development, results thus far have been promising, and suggest it might assume a place in the oncologic armamentarium, at least in an adjuvant capacity. For example, a recent phase III randomized trial (*n* = 436) evaluated the efficacy of talimogene laherparepvec (T-VEC), a granulocyte macrophage colony-stimulating-factor (GM-CSF)-secreting virus based on herpes simplex virus-1. GM-CSF is a glycoprotein white blood cell growth factor that facilitates the maturation and activation of macrophages and dendritic cells. In advanced MM patients, T-VEC injections yielded a higher objective response rate (26% vs. 6%), complete response rate (11% vs. 1%), and durable response rate (a continuous response for at least 6 mo, 16% vs. 2%) compared to GM-CSF alone (*p* < 0.0001), with a trend towards higher OS (HR 0.79, 95% CI: 0.61–1.01) [68]. While serious ADRs were more common in the V-TEC cohort (26% vs. 13%), those grade III and above occurred in less than 3% of either treatment arm. A phase I/II randomized study of T-VEC as an adjuvant to anti-CTLA-4 therapy is recruiting (NCT01740297).

In a similar vein, an ongoing one-armed phase II trial (*n* = 40) is investigating the use of intralesional Coxsackievirus A21 in unresectable stage III-IV MM patients (NCT01227551). Preliminary data show a comparable objective response rate of 24%, and a 35% PFS at 6 months [69]. No grade 3–4 ADRs have been reported. Promising preclinical data have also arisen from use of a modified, co-stimulatory molecule-expressing, IFN-inducing avian Newcastle Disease Virus in conjunction with immune checkpoint blockade therapy [70], and from an adenovirus expressing RNA molecules aimed at reversing the epigenetic silencing of apoptotic programs often present in melanoma [71].

## 3. Esophageal Cancer

Esophageal cancer is the 8th most common cancer worldwide, and is divided into two main types, squamous cell carcinoma, which has a higher incidence in the eastern world, and adenocarcinoma, which comprises a greater and increasing proportion of esophageal cancer in the US [72]. In addition to the traditional oncologic treatment triad of surgery, chemotherapy, and radiation therapy, trastuzumab, an anti-HER2/neu receptor (HER2R) mAb, has provided a survival benefit and gained FDA approval for combined use with chemotherapy in a subset of patients with HER2R-positive gastroesophageal junction cancer. Despite treatment, five-year survival for patients with esophageal cancer post-resection ranges from 20–30% [73].

### 3.1. Anticancer vaccines

Multiple small phase I trials evaluating peptide vaccines as experimental mono- and adjuvant therapy have been conducted in patients with advanced esophageal squamous cell carcinoma. In one 10-patient study, peptide specific immune responses were detected in 9 subjects. One patient with hepatic metastases experienced a complete response lasting 7 months, while three more demonstrated stable disease for at least 2.5 months [74]. In another, combination peptide vaccine and chemoradiation therapy in 11 patients with unresectable disease yielded a peptide-specific cytotoxic T cell response in all subjects, a complete response in 54.5% (6/11), and prolonged CRs of 2–4.6 yrs in a subset who continued vaccinations [75]. Future directions include combining peptide vaccines with chemotherapy and IL-2 (phase II, NCT01795976), and using tumor lysate vaccines in the adjuvant setting with or without chemotherapy (phase I/II, NCT02054104).

### 3.2. Adoptive cell transfer

Only one phase I/II clinical trial to our knowledge has been conducted investigating the use of ACT in esophageal malignancy [76]. In it, peripheral blood mononuclear cell (PBMC)-derived T cells exposed to tumor cells were injected intralesionally, and into metastatic lymph nodes, pleural and peritoneal spaces along with IL-2, yielding complete or partial responses in 36.4% (4/11). In light of preclinical evidence of synergy between DC therapy and RT [77], randomized trials are currently evaluating the use of dendritic and cytokine-induced killer (DC-CIK) cell therapy in combination with RT and chemoradiation therapy (CRT) compared to RT and CRT alone in localized and advanced esophageal cancer (NCT01691664, NCT01691625, respectively). Other avenues of research involve a phase I assessment of tumor antigen (MAGE-A4) gene transduced T cell ACT in unresectable esophageal cancer (NCT02096614), and a phase II trial of cancer testis antigen (NY-ESO-1) gene transduced T cell ACT in combination with low-dose IL-2 in advanced esophagogastric cancer (NCT01795976).

## 4. Gastric Cancer

Gastric cancer is the third most common cause of cancer-related death worldwide, and due to lack of readily available screening tests, is generally advanced at diagnosis with poor (< 20%) 5-year survival among those with metastatic disease [78],[79]. Gastric cancer generally stems from chronic atrophic gastritis secondary to H. pylori bacterial infection, which sets the stage for mucosal metaplasia and ultimately malignancy. Prevention ideally involves treatment of any underlying H. pylori infection. Inroads in immunotherapy have yielded promising new agents on the horizon for gastric cancer treatment to complement the current armamentarium of surgery, chemotherapy, and radiation, and, more recently, multiple FDA-approved mAbs against vascular endothelial growth factor (VEGF, ramucirumab, 2014) and HER2/neu receptors (trastuzumab, 2010), which offer survival benefits in certain subgroups [80],[81].

### 4.1. Anticancer vaccines

Tumor vaccination strategies have ranged from the use of peptides to live attenuated pathogens, DCs and CIK cells pulsed with peptides or cell lysates, and gene therapy, largely in the adjuvant setting. Peptide vaccines have shown survival benefits both alone and in combination with chemotherapy. For example, a phase II trial of a peptide vaccine designed to elicit an anti-gastrin-antibody-mediated anti-tumor immune response in 94 patients with untreated, metastatic or unresectable gastric or gastroesophageal adenocarcinoma demonstrated an ORR of 30%, and significantly greater median PFS (5.5 mo vs. 2.2 mo, *p* = 0.0005) and OS (10.3 mo vs. 3.8 mo, *p* = 0.0001) in immune-responders (65/94, 69%) than non-responders, with minimal ADRs [82]. Similarly, a phase I/II trial of S-1 (peptides derived from VEGF receptors) administered with chemo in 22 patients with advanced or recurrent gastric adenocarcinoma yielded a PR of 55%, and a median OS of approximately 2.5 years in immune-responders (18/22, 82%), more than double that of their non-responding counterparts (*n* = 4, *p* = 0.028) [83].

Adjuvant immunochemotherapy with the nearly century-old live attenuated Mycobacterium bovis bacillus Calmette-Guerin (BCG) vaccine likewise conferred a 10-year survival benefit (47.1%, *p* < 0.037 vs. chemo, *p* < 0.0006 vs. control) in 156 patients with stage III-IV gastric cancer post-curative surgical resection compared to adjuvant chemo (30%) and no adjuvant therapy (15.2%) [84]. Patients who benefited most were those with primary T2/T3 tumors and intestinal type tumors, whereas those with diffuse-type cancer did not benefit. A DC vaccine pulsed with HER2/neu peptides yielded modest results in a small phase I trial [85], and a phase II randomized study investigating autologous tumor lysate-pulsed DC & CIK cells in the adjuvant setting in stage I–III gastric cancer is currently recruiting (NCT02215837). Another intriguing avenue of research involves HER2 gene therapy via a modified equine encephalitis virus in metastatic, HER2R+ malignancies including gastric cancer (phase I, NCT01526473).

### 4.2. Adoptive cell transfer

ACT, with TILs, CIK cells, and NK cells, has been investigated in gastric cancer with varying degrees of success in the adjuvant setting. For example, in a randomized trial of 44 patients with stage IV gastric cancer, TIL-based ACT with chemotherapy yielded significantly greater 50% survival rates than chemo alone (11.5 vs. 8.3 mo, *p* < 0.05) [86]. Placed head-to-head with chemotherapy, CIK cell ACT yielded longer median survival (49 mo vs. 27 mo, *p* < 0.05) and greater 5-year OS (40.4% vs. 23.9%, *p* < 0.05) than chemo in 156 post-op gastric cancer patients [87]. Frequency of re-infusion correlating to decreased risk of death (HR 0.54, 95% CI: 0.36–0.80). Shi et al. 2012 found similar results comparing adjuvant CIK ACT to no adjuvant therapy in post-op patients with stage III-IV gastric cancer, though the 5-year OS difference proved statistically significant only for the subgroup with intestinal-type tumors (46.8 vs. 31.4%, *p* = 0.045) [88].

Future directions include combining TIL ACT with HD IL-2 and chemotherapy (phase II, NCT01174121), combining anti-carcinoembryonic antigen (CEA)-specific autologous T cells with IL-2 in CEA+ metastatic gastric, colorectal, and lung cancers (phase II, NCT01723306), combining NK cell ACT with anti-HER2/neu biologic therapy in HER2R+ gastric cancers (phase I/II, NCT02030561), and assessing DC and CIK cell ACT in combination with chemotherapy for recurrent or metastatic gastric or esophagogastric junctional adenocarcinomas (phase I/II, NCT01783951).

### 4.3. Immunomodulatory mAbs

Checkpoint modulation in gastric cancer is off to an encouraging start, both in the monotherapy and adjuvant settings. Over an impressive 15-year follow-up period, a phase III trial comparing chemoimmunotherapy with dsRNA TLR agonist polyadenylic-polyuridylic acid with chemo alone in 292 post-op gastric cancer patients showed prolonged 15-yr PFS (59.4 vs. 44.1%, *p* = 0.005) and OS (50.1 vs. 38.1%, *p* = 0.013) with chemoimmunotherapy [89]. In an ongoing phase I trial, anti-PD-1 therapy with pembrolizumab (10 mg/kg q2W up to 24 mo) in PD-L1 expressing gastric cancer (*n* = 39) with median 6 mo follow-up has so far yielded an ORR of 30–32%, and a median response duration has not yet been reached (range 2–5 mo, NCT01848834) [90]. PD-L1 expression correlated with PFS and ORR, and grade 3–4 ADRs occurred in 7.7%. A phase I multicenter study of the anti-PD-L1 mAb MEDI4736 in multiple solid tumors, including gastric cancer, is also underway (NCT01693562).

Ertumaxomab (ertu), another mAb, is designed to form a tri-cell complex with HER2/neu+ tumor cells, CD3+ T cells, and accessory cells in order to potentiate direct, and immune-mediated anti-tumor responses [91]. A phase I/II dose-escalation trial is currently evaluating ertu in patients with a variety of solid tumors including gastric cancer, so far with encouraging safety results and signs of immune response (NCT01569412) [92]. Based on a) initial promising immune responses to adjuvant catumaxomab, another trifunctional (anti-EpCAM and -CD3) mAb, in the setting of surgery and RT in patients with non-metastatic gastric cancer [93], and b) modestly improved OS with catumaxomab in the setting of gastric peritoneal carcinomatosis (PC, *n* = 66; 71 vs. 44 days, *p* = 0.031) [94], a phase II study is now assessing 2-yr OS with surgery and adjuvant catumaxomab in patients with gastric PC (NCT01784900).

Other active avenues of research include comparing anti-CTLA-4 therapy to supportive standard of care following first-line chemo in patients with unresectable locally advanced or metastatic gastric and gastroesophageal junction malignancy (phase II, NCT01585987), and evaluating anti-PD-1 therapy with or without anti-CTLA-4 blockade in multiple solid tumors, including gastric cancer (phase I/II, NCT01928394). Future potential targets in gastric cancer include T regs, myeloid-derived suppressor cells (MDSCs), and bone-marrow derived mesenchymal stem cells (BM-MSCs), all of which have been shown to promote an immunosuppressive environment in the setting of gastric cancer, as well as secreted immunosuppresive molecules (e.g. TGF-b, IL-10) that are associated with reduced survival in this disease [95]–[97].

## 5. Pancreatic Cancer

Pancreatic cancer is the fourth most deadly cancer in the United States, and pancreatic ductal adenocarcinoma comprises 90% of cases. With a dismal five-year survival rate (overall < 6%, and < 20% in resectable disease), and increasing incidence (the number of pancreatic cancer cases is estimated to double by 2030), an urgent need exists for improved therapies [98],[99]. Complete surgical resection is the only curative treatment for pancreatic cancer, however, less than 20% of patients are candidates for surgical resection, as the cancer often remains undetected until it reaches an advanced stage. Immunotherapy for pancreatic cancer provides a promising complement to current treatment options, though none have yet received FDA approval [100].

### 5.1. Anticancer vaccines

A variety of cancer vaccines have been experimentally applied to pancreatic cancer, including tumor-associated antigen vaccines and cell-based vaccines. Cell-based vaccines may be autologous or allogeneic, and they utilize whole cells as the source of multiple antigen targets, while tumor-associated antigen vaccines target specific antigens characteristic of tumor cells. Despite promising phase I/II clinical trial data for DC and peptide-based vaccines targeting tumor-associated antigens MUC1 [101] and telomerase [102], phase III trial outcomes have failed to demonstrate survival benefits over chemotherapy alone for the adjuvant treatment of advanced pancreatic cancer (APC) [103],[104]. Phase I/II evidence suggests peptide vaccines based on kinesin proteins that are transactivated in pancreatic cancer may confer a survival benefit in patients with chemo-refractory APC compared to historical controls [105], warranting further investigation.

Tumor mutations represent another potential antigenic vaccine target. KRAS mutations are present in 90% of pancreatic adenocarcinomas. In a phase I/II clinical trial, an intradermal combination of KRAS mutant-derived synthetic peptides and GM-CSF administered to 48 pancreatic adenocarcinoma patients showed a peptide-specific immune response in 58% (25/43) of evaluable patients, and a prolonged median OS in this subset compared to immune non-responders (148 vs. 61 days, *p* = 0.0002) [106]. Another long-term phase I/II study demonstrated that 85% (17/20) of post-op patients with RAS-mutated pancreatic adenocarcinomas developed an immune response to a RAS-specific peptide vaccine, and 20% (4/20) survived at least a decade, three quarters of whom maintained an immune response for up to 9 years after initial vaccination [107]. Further studies evaluating RAS peptide-based vaccines are warranted.

Allogeneic pancreatic tumor cells engineered to secrete GM-CSF and then irradiated prior to injection (GVAX) represent another tumor vaccination strategy. A phase I trial utilizing this approach in 14 patients with resected stage I-III pancreatic cancer, 86% (12/14) of whom went on to receive adjuvant CRT [108]. Twenty-one percent (3/14) experienced delayed-type hypersensitivity responses to their autologous tumor cells and seemed to show increased RFS of at least 25 months post-diagnosis. A phase II trial studying the same vaccine approach in combination with CRT in a similar patient population (*n* = 60) showed a median RFS of 17.3 months and median OS of 24.8 months [109], and another similar trial is ongoing (NCT01595321). Preliminary results from another phase II study demonstrated a survival benefit with a combination of low-dose cyclophosphamide (to inhibit T regs), GVAX, and a live-attenuated *Listeria monocytogenes* vaccine (CRS-207) versus GVAX alone in 90 patients with metastatic colon cancer (8.2 vs. 4.0 mo, HR = 0.23, *p* = 0.0003) [110], prompting a phase IIb randomized controlled trial comparing GVAX and CRS-207 to chemotherapy or CRS-207 alone in patients with pre-treated metastatic pancreatic adenocarcinoma (NCT02004262).

Similarly, algenpantucel-L immunotherapy consists of allogeneic pancreatic tumor cells modified to express a carbohydrate (α-Gal) to which humans have natural immunity. A recent 71-patient phase II study of combination algenpantucel-L and CRT for resected pancreatic cancer showed a positive correlation between presence of a specific immunologic response as evidenced by an elevated anti-calreticulin antibody titer, seen in 48%, and prolonged median OS (35.8 vs. 19.2 mo, *p* = 0.03) [111]. Follow-up phase III studies are underway (NCT01836432, NCT01072981).

Finally, antibody-drug conjugates like the tumor-associated antigen-binding mAb-topoisomerase inhibitor IMMU-132 (isactuzumab govitecan), represent another innovative line of anti-cancer immunotherapeutic research, with promising preliminary phase I/II results in a variety of metastatic GI cancers including those of esophageal, gastric, pancreatic, and colorectal origin (NCT 01631552) [112].

### 5.2. Adoptive cell transfer

Adoptive cell therapy has not been thoroughly investigated for pancreatic cancer. One study, investigating the combination of MUC1-specific DC therapy in combination with MUC1-specific cytotoxic T lymphocyte ACT in 20 patients with advanced pancreatic cancer (APC) yielded a CR in one patient (5%), stable disease in 25% (5/20), a mean survival of 9.8 mo, and no grade 3–4 toxicity [113]. A recent phase II trial demonstrated a 25% disease control rate in 16 patients with chemo-refractory APC, and while no survival benefit was suggested over chemotherapy compared to historical data, ACT seemed to confer a quality of life benefit [114]. A phase I/II trial evaluating DC-CIK ACT in combination with chemotherapy in APC (NCT01781520), and a phase II trial investigating TIL ACT in combination with HD IL-2 in APC and other GI cancers (NCT01174121) are recruiting.

### 5.3. Immunomodulatory mAbs

Given its significant survival benefit in subsets of metastatic melanoma patients, the anti-CTLA-4 antibody, ipilimumab, has been studied in pancreatic ductal adenocarcinoma patients as well. A phase II trial evaluating ipilimumab (3 mg/kg q3W × 4 doses/course for a maximum of 2 courses) in 27 patients with advanced pancreatic cancer, found the monotherapy ineffective for this disease with only one patient displaying a delayed PR after initial disease progression [115]. A more recent randomized, 30-patient trial comparing ipilimumab (10 mg/kg) with a GM-CSF vaccine to ipilimumab alone in a similar patient population suggested a trend toward higher median OS in the combination therapy cohort (5.7 vs. 3.6 months, HR = 0.51, *p* = 0.072), and higher 1-year OS (27% vs. 7%), with a 20% incidence of grade 3–4 ADRs in both arms [116]. A corresponding phase II trial evaluating the efficacy of chemotherapy followed by combination ipilimumab and GM-CSF vaccine is recruiting (NCT01896869).

Other immune checkpoint modulation approaches in APC involve anti-PD1 therapy with pembrolizumab and CD40 agonism with CD-870873. The latter yielded a 19% response rate in combination with chemotherapy in a small, preliminary trial of 21 patients with APC [117]. The efficacy of anti-PD-1 therapy with pembrolizumab in conjunction with neo-adjuvant CRT compared to CRT alone in APC will be explored in an upcoming phase I/II trial (NCT02305186).

## 6. Colorectal Cancer

Colorectal cancer (CRC) is the third-most common cancer worldwide [118]. Screening tests, such as stool guaiac tests and colonoscopies, can aid in the early diagnosis of CRC, at which stage 5-year survival is a favorable 90%. Less than half of CRCs, however, are diagnosed at such a stage, and the presence of metastatic disease reduces the 5-year survival rate to 69% [119]. Surgical resection is the most effective curative treatment for localized CRC, however, 50% of these patients develop recurrence [120]. Just as chemotherapy may deliver a survival benefit in metastatic CRC, so too can immunotherapy in certain subsets of patients. Current investigative approaches include cancer vaccination, adoptive cell therapy, and immune checkpoint modulation.

### 6.1. Anticancer vaccines

As in pancreatic cancer, experimental tumor vaccines in CRC utilize “self” tumor-associated antigens (i.e. antigens normally expressed by certain cells that are differentially overexpressed and thus targetable in tumor cells), “non-self” (i.e. mutation-based) tumor-associated antigens, and whole cells. “Self” antigens have included Carcinoembryonic Antigen (CEA), for which the presence of corresponding autoantibodies in CRC patients correlates with better prognosis and increased 2-year survival rates [121], Epithelial Cell-Adhesion Molecule (Ep-CAM), and mucin glycoprotein MUC1, among others, all of which are overexpressed by subsets of CRC tumor cells. Despite eliciting immune responses in subsets of patients, most of the corresponding vaccines have yet to demonstrate a survival benefit [122]–[124].

A phase I study, however, evaluating the combination of five HLA-A (human leukocyte antigen-A) antigens, each subcutaneously injected at separate sites in 18 HLA-A*2402-positive CRC patients, demonstrated a CR in one patient, stable disease over 4–7 months in six others, and a median OS of 13.5 months [125]. Patients who experienced a specific immune response to 3 or more of the peptides enjoyed a significantly prolonged OS compared to immune non-responders (27.3 mo vs. 3.7 mo, *p* = 0.032). Given minimal ADRs, this approach warrants further investigation.

Another treatment that has shown preliminary promise involves gene therapy via plasmid DNA encoding truncated human CEA fused to a T-helper epitope (CEA66 DNA). When delivered intradermally (2 mg) or intramuscularly (8 mg) with GM-CSF after cyclophosphamide treatment to 10 resected CRC patients, 80% showed no evidence of recurrence at a median 72-week follow-up [120]. No grade 3 or 4 adverse events were reported.

“Non-self” tumor associated antigens previously targeted by experimental CRC vaccines include mutant p53 and RAS proteins. In a study evaluating a mutant p53-exposed PBMC-based vaccine in 24 patients with malignant p53-mutated tumors (including 10 CRC patients), 45% (9/20) of evaluable patients experienced a detectable cellular immune response and PFS and OS were greater than expected (12.5 and 27.2 months, respectively) [126]. Similarly, in a phase I trial of a RAS-mutant-specific peptide vaccine in RAS-mutated cancer patients (5 pancreatic cancer and 7 CRC patients), 45% of evaluable patients (5/11) experienced a detectable immune response [127]. The CRC patients had a RFS of 27.2 months and OS of 41.5 months.

Regarding whole-cell-based vaccines, a meta-analysis analyzing three phase III, randomized trials of OncoVAX (autologous colorectal tumor cells with adjuvant BCG vaccine) for resected stage II-III CRC showed no OS benefit, though RFS was prolonged for stage II colon cancer patients, and their 5-year recurrence rate was significantly lower than that of controls (21.3% vs. 37.7%, *p* = 0.009) [128],[129]. Another cell-based vaccine method utilizes modified virus-infected tumor cells. A phase III trial evaluating a modified Newcastle disease virus (NDV)-infected autologous tumor cell vaccine in 50 patients with resected metastatic CRC demonstrated longer metastasis-free and OS in the vaccinated cohort (HR 2.7, 95% CI: 1.0–7.4, *p* = 0.042, and HR 3.3, 95% CI: 1.0–10.4, *p* = 0.047, respectively) [130].

### 6.2. Adoptive cell transfer

Similar to pancreatic cancer, investigations of ACT in CRC have been limited. A small study assessing murine anti-CEA receptor-engineered T cells in three patients with refractory metastatic CRC showed significant reduction in serum CEA levels in all patients and an objective response in one of the three patients, though all three experienced severe, transient inflammatory colitis [131]. Another, utilizing autologous lymphocytes from tumor-draining sentinel lymph nodes in 16 patients with metastatic CRC, showed a complete response in 4 of 9 patients and no serious ADRs [131]. A trial evaluating anti-VEGFR2 genetically-modified cytotoxic TILs in a variety of metastatic cancers, including CRC, is currently recruiting (NCT01218867).

### 6.3. Immunomodulatory mAbs

Neither anti-CTLA-4 therapy, nor anti-PD-1 therapy have garnered substantial evidence of clinical benefit in CRC thus far [132]–[134]. For example, a small (*n* = 18), multicenter, dose-escalation phase I trial of anti-PD-L1 antibody in CRC yielded no objective responses [58]. A phase I trial of nivolumab (3 mg/kg) administered to one CRC patient, however, resulted in a complete response with no recurrence in the 3 years since [134]. Further trials assessing checkpoint inhibitors and their combinations are underway. For example, trials evaluating a combination of the anti-PD-L1 and anti-CTLA-4 antibodies MEDI4736 and tremelimumab (NCT01975831), as well as combination PD-1 inhibition and RT (NCT02298946) are recruiting.

## 7. Lung Cancer

Pulmonary malignancies are divided into two broad categories: small cell lung cancers (SCLC) and non-small cell lung cancers (NSCLC). NSCLC is more common and occurs in about 90% of the population while SCLC only occurs in about 10% of the population. In the past decade, lung cancer treatments have generally focused on targeted therapies such as epidermal growth factor receptor (EGFR) tyrosine kinase inhibitors and anaplastic lymphoma kinase (ALK) fusion protein inhibitors. Unfortunately, 5-year OS for lung cancer remains less than 20% [135]. Though originally thought to be a poorly immunogenic tumor subtype, recent studies have shown that immunotherapy can induce clinically relevant responses in this disease. As of 2014, there are no FDA-approved immunotherapies for lung cancer, though clinical trials are currently assessing the efficacy of vaccines, immune checkpoint inhibitors, and various immunotherapy combinations.

### 7.1. Anticancer vaccines

TG4010 is a cancer vaccine based on a modified viral vector expressing tumor-associated antigen MUC1 and IL-2. In a phase IIb study, 217 NSCLC patients received TG4010 combined with first-line chemotherapy. Preliminary results showed a statistically significant improvement in PFS for non-squamous NSCLC patients treated with TG4010 [136]. The phase 3 trial for TG4010 is currently recruiting (NCT01383148).

### 7.2. Immunomodulatory mAbs

#### 7.2.1. CTLA-4 inhibition

Anti-CTLA-4 therapy has shown some efficacy in melanoma tumors and is being investigated in lung cancer. In a randomized, double-blind, multicenter phase II study, 204 chemotherapy-naïve NSCLC patients were randomized to three groups: control (only chemotherapy), concurrent ipilimumab (chemotherapy and ipilimumab concurrently), and phased ipilimumab (chemotherapy followed by ipilimumab) [137]. Phased ipilimumab, concurrent ipilimumab, and control treatments had a median PFS of 5.1, 4.1, and 4.2 months, respectively. Overall rates of grade 3/4 immune-related AEs were 15%, 20%, and 6% for phased ipilimumab, concurrent ipilimumab, and the control, respectively. Ipilimumab is now being further tested in two phase III trials for NSCLC (NCT01285609) and SCLC (NCT01450761).

#### 7.2.2. PD-1 inhibition

The efficacy of anti-PD-1 agents nivolumab and pembrolizumab is also under investigation in lung cancer. A phase 1 trial evaluated the response of 76 NSCLC patients to nivolumab at dosages: 1 mg/kg, 3 mg/kg, or 10 mg/kg. Eighteen percent (14/76) of NSCLC patients responded [138]. Follow up on nivolumab-treated patients revealed a 2-year OS rate of 24% and median OS of 9.9 months [139]. Based on these results, two phase III trials are comparing nivolumab to chemotherapy in stage IV squamous and non-squamous cell NSCLC (NCT01642004, NCT01673867).

Another phase I trial assessed pembrolizumab (2mg/kg Q3W, 10mg/kg Q3W, or 10mg/kg Q2W) in 262 NSCLC patients [140]. The ORR for all NSCLC was 21%. ORR was higher in patients with ≥ 1% PDL-1 staining (23% vs. 9%). Grade 3–5 drug-related adverse events occurred in 9% of patients. Multiple trials are currently evaluating pembrolizumab in advanced lung cancer alone (NCT02007070), in comparison to chemotherapy (NCT02220894, NCT01905657), and in combination with chemotherapy (NCT01840579) and ipilimumab (NCT02039674).

#### 7.2.3. PDL-1 inhibition

Experimental PD-L1 inhibitors BMS-936559, MPDL3280A, and MEDI4736 have all been evaluated in lung cancer. In a dose-escalation phase I trial, BMS936559 was administered to 75 NSCLC patients, yielding an ORR of 10.2% (5/49 patients) [58]. As of 2014, clinical development of BMS-936559 has been suspended [141]. Another phase I trial assessed MPDL3280A in 53 heavily pre-treated NSCLC patients [142]. Of the 37/53 patients available for evaluation, 24% (9/37) experienced an objective response, with response duration ranging from 1–214 days (7.1 mo), and 24-week PFS was 46%. Grade 3–4 ADRs occurred in 34% of patients. Multiple phase I–III trials are currently evaluating MPDL3280A alone (NCT01846416, NCT01375842, NCT02031458) and in comparison to chemotherapy (NCT01903993, NCT02008227) in lung cancer.

A third phase I trial evaluated escalating doses of MEDI4736 Q2W or Q3W in 13 NSCLC patients [143]. Preliminary results suggest a PR rate of 23.1% (3/13 patients). Multiple phase I–III clinical trials are further assessing the efficacy of various regimens of MEDI4736 (NCT02087423, NCT01693562, NCT02125461).

#### 7.2.4. Combination therapy

A recent phase I trial assessed nivolumab in combination with ipilimumab in 46 chemotherapy-naïve lung cancer patients. Preliminary results show a 22% ORR and a 48% incidence of grade 3–4 ADRs [144]. Another early phase I/II trial investigating the same combination in SCLC patients is recruiting (NCT01928394), and multiple other clinical trials are likewise evaluating the combination of anti-CTLA-4 and anti-PD-L1 therapy in lung cancer patients (NCT02261220, NCT02000947, NCT01975831).

## 8. Summary and Future Prospects for Cancer Immunotherapy

The last few decades have witnessed an accelerating resurgence of the quest to utilize the body's own immune system to more efficiently and effectively identify and combat cancer with minimal damage to healthy tissue. A number of strategies have arisen in this pursuit and a series of breakthroughs, most notably immune checkpoint modulating mAbs, have conferred a significant survival benefit in subgroups of patients with a variety of solid tumors, including melanoma and gastric cancer. Numerous avenues of clinical investigation are underway to evaluate the efficacy of immunotherapies as single agents, in combination therapy, and in the adjuvant setting for these malignancies as well as those of the rest of the GI tract, lungs, and other organ systems. Identifying and validating new potential immune checkpoint targets, as well as honing the engineering of exogenously expanded autologous immune effector cells to more selectively target tumors while minimizing collateral damage to the body represent major ongoing focuses of research. Due to the significant differential benefits and adverse effects seen in subsets of immunotherapy patients, efforts to uncover convenient biomarkers, from serum assays to immunohistochemical tumor microenvironment and immune effector cell profiles, associated with favorable outcome-to-adverse event balances in the case of each therapeutic, remains one of the most challenging and necessary gaps to bridge in this field. Identifying and characterizing these outcome predictors will greatly facilitate the selection of appropriate patients for treatment, thereby maximizing therapeutic efficacy and the efficient allocation of healthcare resources, minimizing harm, and aiding our march forward into the era of increasingly personalized medicine.
